# Supplementary material for: Amelioration of Congenital Tufting Enteropathy in EpCAM (TROP1)-Deficient Mice via Heterotopic Expression of TROP2 in Intestinal Epithelial Cells
Source: Cells. 2020 Aug 6;9(8):1847. doi: 10.3390/cells9081847 (PMC7465201; doi:10.3390/cells9081847)
Supplement: Supplementary file 1 [file cells-09-01847-s001.pdf]

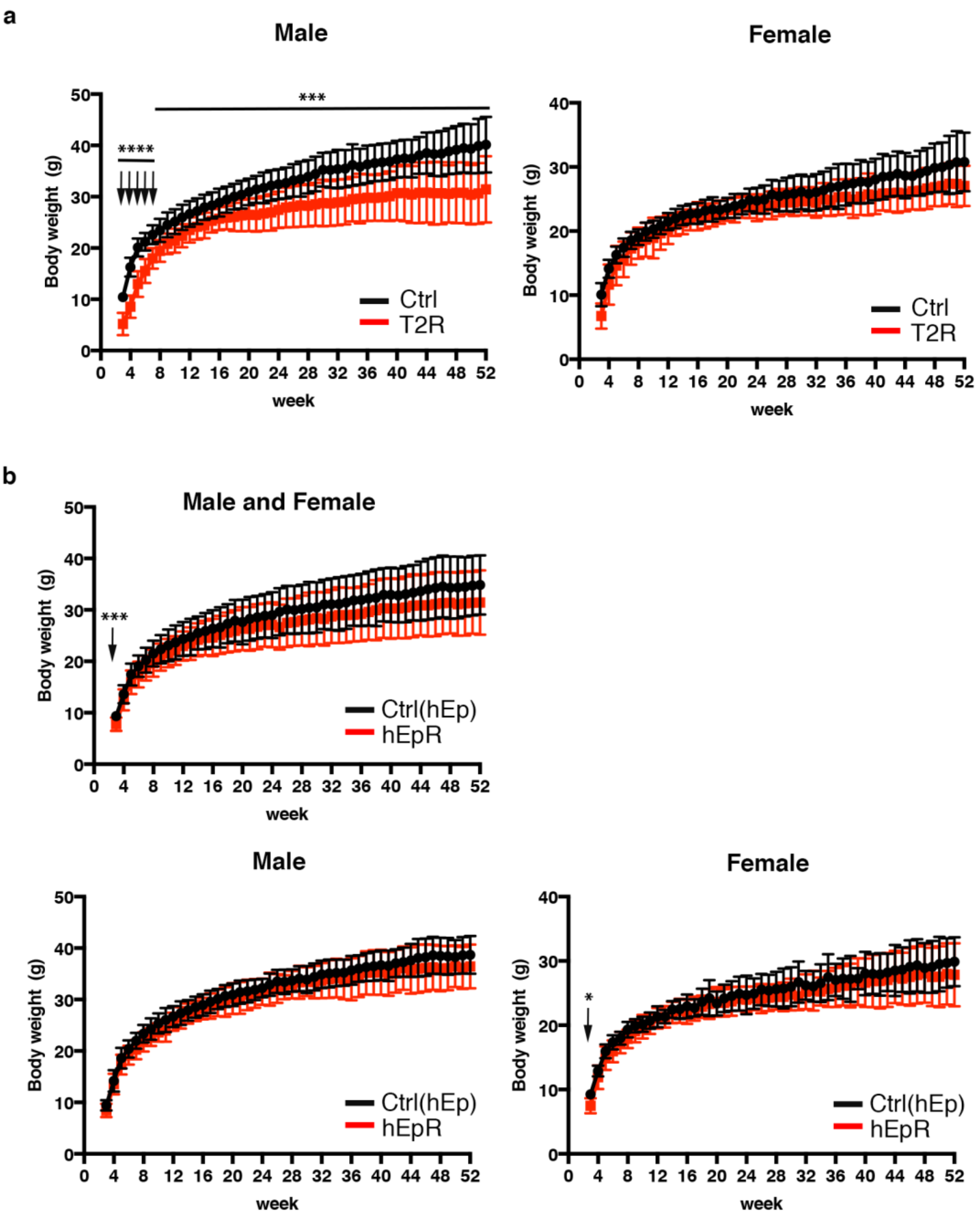

Figure S1. Sex differences in body weights of TROP2 rescue mice. (**a**) Serial body weights of male (left panel) and female (right panel) littermate control [$EpCAM^{+/-}$ $TROP2^{Tg}$; Ctrl (T2)] and TROP2 rescue mice obtained over 1 year. We measured body weights of 32 littermate control mice (male:17, female: 15) and 24 T2R (male:13, female: 11). Data are expressed as means ± SD. * $p < 0.05$ *** $p < 0.001$, **** $p < 0.0001$ as determined using a multiple t test with the Holm-Sidak method. (**b**) Serial body weights of littermate control [$EpCA^{+/-}$ $hEpCAM^{Tg}$; Ctrl (T2)] and human EpCAM rescue mice obtained over 1 year. Data depicted represent means ± SD for 23 (male:13, female: 10) Ctrl mice and 24 (male:10 and female:14) hEpR mice at each time point. Data are expressed as means ± SD. * $p < 0.05$ *** $p < 0.001$ as determined using a multiple t test with the Holm-Sidak method.

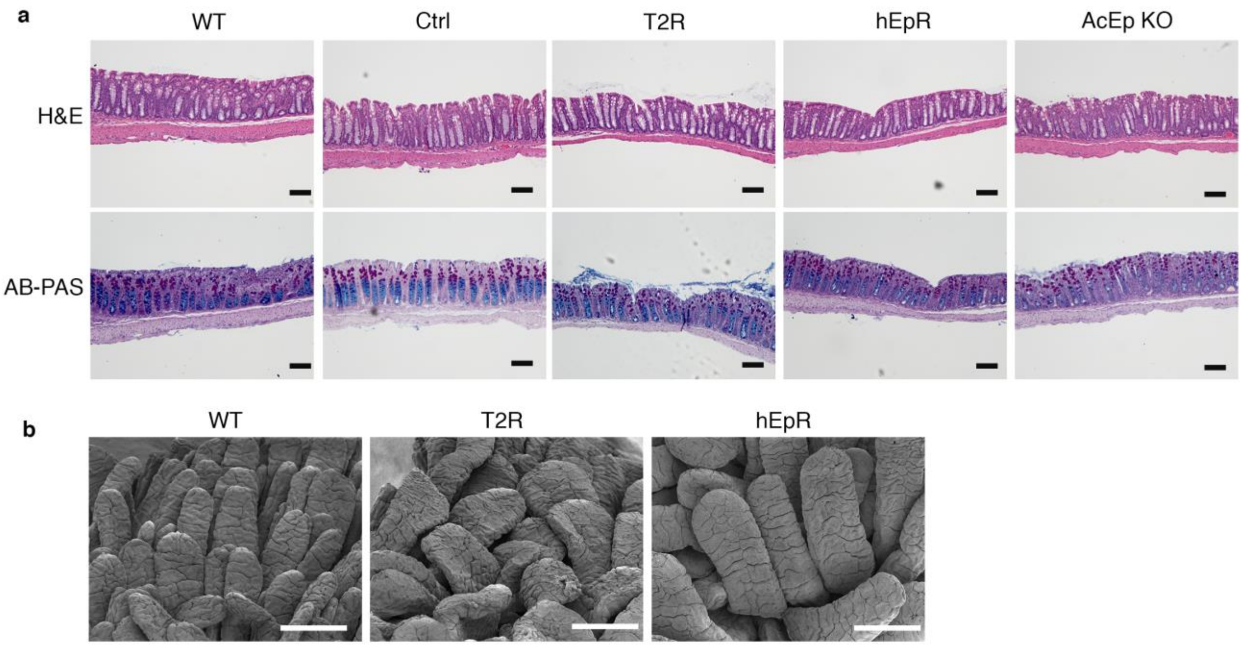

Figure S2. Additional characterization of TROP2 rescue IEC. (**a**) H&E and Alcian blue-PAS staining of sections of fixed tissue from colons of indicated mice. Scale bars, 100 μm. (**b**) Surface structure of small intestinal villi of WT, T2R and hEpR mice revealed by scanning electron microscopy. Scale bars, 200 μm.

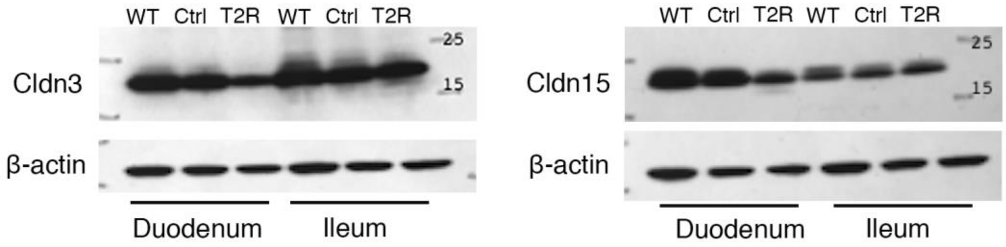

Figure S3. Additional characterization of claudin expression in TROP2. Tissue lysate proteins from duodenums and ilea were resolved using SDS-PAGE and immunoblotted with anti-claudin-3, anti-claudin-15 and anti-β-actin as indicated.

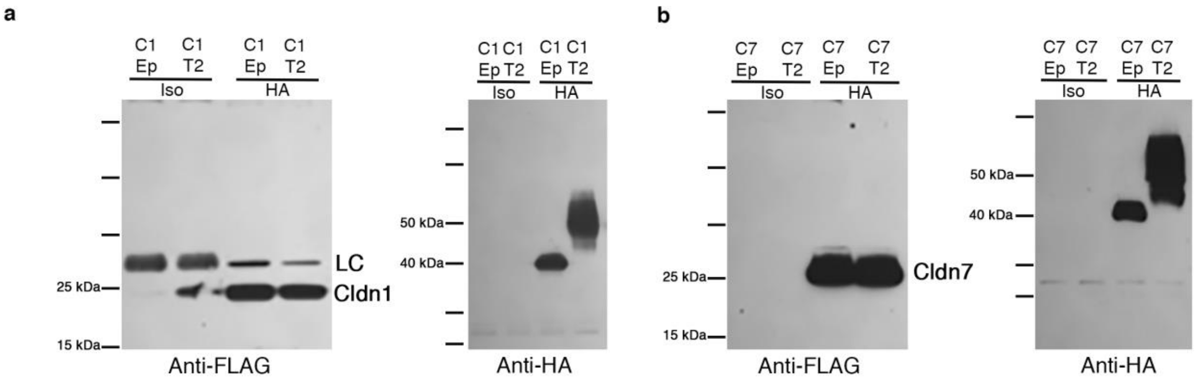

Figure S4. Association of claudin-1 and claudin-7 with EpCAM and TROP2. (**a**) HEK 293T cells transfected with plasmids encoding Flag-claudin-1 and HA-EpCAM or HA- TROP2 were lysed and immunoprecipitated with control IgG (Iso) or rat anti-HA (HA). Immunoprecipitates were resolved by SDS-PAGE and serially immunoblotted with anti-FLAG and rabbit anti-HA as indicated. (**b**) HEK 293T cells transfected with plasmids encoding Flag- claudin-7 and HA-EpCAM or HA-TROP2 were lysed and immunoprecipitated with control IgG (Iso) or rat anti-HA (HA). Immunoprecipitates were resolved by SDS-PAGE and serially immunoblotted with anti-FLAG and rabbit anti-HA as indicated. C1, claudin-1; C7, claudin-7 ; Ep, EpCAM ; LC, light chain ; T2, TROP2.

Supplementary Methods

Verification of transgenes by PCR genotyping.

Transgenic mice carrying mVillin-mTrop2 or mVillin-hEpCam were identified by PCR genotyping of tail DNA using specific primers (Supplementary Table 1). Mouse tail lysates were prepared using Viagen mouse tail lysis buffer and proteinase K following manufacturer's instructions and then 1 μL of template was added to 2X DreamTaq Green PCR Master Mix with water to a total reaction volume of 25 μL. After a 5 min 95 °C denaturation step, PCR amplification was carried out for 30 cycles at 95 °C for 30 s, 60 °C for 30 s, 72 °C for 1 min.

Verification of EpCAM deletion by PCR genotyping.

Germline EpCAM allelic deletion in the heterozygous state was verified by genotyping pup tail lysates for the presence of the wild-type EpCAM band and the complete absence of the floxed EpCAM band using the $mEpCAM^{fl/fl}$ primers set. An independent PCR confirmation of a positive 371 bp deletion product was verified by using the $EpCAM^{del}$ primer set. The cycling conditions were: 95 °C for 3 min followed by 30 cycles of 95 °C for 30 s, 60 °C for 30 s, 72 °C for 1 min.

Generation of anti-murine EpCAM and anti-TROP2 antibody.

Rabbits were immunized and boosted subcutaneously several times with recombinant, affinity-purified proteins comprised of the extracellular portions of murine EpCAM or murine TROP2 fused in frame to the Fc portions of human IgG1 in Titermax (Titermax, Norcross, GA, USA). Immunization was verified by ELISA measurement of antibody in peripheral blood, and splenic B cells were fused with non-producing rabbit myeloma cells. Hybridoma sub-clones were isolated by limiting dilution in 96 well plates and individually collected supernatants were tested by ELISA for positive reactivity to the original immunogen and negative reactivity to human IgG. Clones E73 (mEpCAM) and T69 (mTROP2 ECD) were

selected for use based on their superior sensitivities in assays that included Western blotting, immunofluorescence, immunohistochemistry and flow cytometry. E73 and T69 were expanded and adapted to bioreactors to generate larger quantities of concentrated antibodies that were captured by affinity column A purification using an AKTA high performance chromatography instrument (GE Healthcare, Pittsburgh, PA, USA). The eluted antibodies were neutralized with final concentration 60 mM Tris-HCl (pH 9), dialyzed and stored in PBS (pH 7.3) at −80 °C prior to use.

RNA-FISH.

Sections were dried at 60 °C for 1 h, and then dewaxed through a series of a xylenes and alcohols. Cellular RNA was made accessible by boiling slides for 15 min in 1 X Target Retrieval Reagent followed by a 30 min incubation at 40 °C with Protease Plus. Transcript-specific probes were designed by ACDBio. Olfm4-C2 probe and Defa1-C1 probe were used at appropriate dilutions. 3-plex negative control probes or Defa-C1/Olfm4 duplex probes were hybridized for 2 h at 40 °C followed by washing with 0.5X Wash Buffer. Signals were amplified using RNAScope Multiplex Fluorescent Detection Reagents V2 (ACD) and the TSA™ Plus Fluorescein (PerkinElmer, Shelton, CT, USA) and TSA™ Plus Cyanine 3 (PerkinElmer) for Defa-C1 and Olfm4-C2 probes respectively following the manufacturer's instructions. Slides were counter-stained with DAPI and cover slipped with ProLong® Gold Antifade Reagent (Life Technologies, Carlsbad, CA, USA). Slides were dried at RT overnight and scanned at 20X magnification using an Aperio ScanScope FL with an Ex:377/±50, Em:447/±60 filter for DAPI counterstain, an Ex:482/±35, Em:536/±40 filter for Fluorescein, and an Ex:543/±22, Em:593/±40 filter for Cyanine 3 visualization.

Table S1. Primer sequences for cloning and genotyping.

Target	Forward Primer (5′-3′)	Reverse Primer (5′-3′)
mTROP2	GCGCCTCGAGATGGCGAGGGGCTTGG	GCGCATCGATCTACAAGCTAGGTTCG
hEpCAM	GCGCCTCGAGGCGCCCCCGCAGGTCC	GCGATCGATTTATGCATTGCGTTCCCTATG
Villin-mTrop2	GCCTGGCTCGACGGCCACTGCTCTCAC A	GTTAGCGTGGAGCAGTCGACCAATACCT
Villin-hEpCam	GCCTGGCTCGACGGCCACTGCTCTC	CTGGCATTGACGATTATTATTCACAAA
mEpCAM$^{fl/fl}$	GCCCAATTCTAGTGGGGAGTGTCATG	GCCTCCAGGCTTTTGTCTCAGATCAG
EpCAM^{del}	GCCCAATTCTAGTGGGGAGTGTCATG	GAAATTCTCTCCAGACCGCTTTG
